# Assessing the potential of longitudinal smartphone based cognitive assessment in schizophrenia: A naturalistic pilot study

**DOI:** 10.1016/j.scog.2019.100144

**Published:** 2019-04-18

**Authors:** Gang Liu, Philip Henson, Matcheri Keshavan, Jukka Pekka-Onnela, John Torous

**Affiliations:** a Department of Biostatistics, Harvard TH Chan School of Public Health, Boston, MA, United States; b Division of Digital Psychiatry, Department of Psychiatry, Beth Israel Deaconess Medical Center, Harvard Medical School, Boston, MA, United States

## Abstract

**Background:**

Although cognition is a core symptom of schizophrenia and associated with functional impairment, the degree of training for and time associated with its assessment makes it difficult to routinely monitor in clinic care.

Smartphone based cognitive assessments could serve as a tool to measure cognition in real time as well as being easily scalable for broad use.

Combined with other data gathered from smartphone sensors such as steps, sleep, and self-reported symptoms – capturing ‘cognition in context’ could provide a powerful new tool for assessing the functional burden of disease in schizophrenia.

**Methods:**

18 participants with schizophrenia and 17 healthy controls completed novel cognitive assessments on their personal smartphones over the course of 12 weeks while also capturing self-reported surveys and step count. No payment or incentives were offered for engaging with the smartphone app. Differing levels of difficulty in cognitive tasks were tested and the results were modeled using a modified Cox proportional hazard model.

**Results:**

On the smartphone cognitive assessments that involved on simple patterns, both controls and those with schizophrenia achieved similar scores. On the more complex assessment that added task switching in addition to pattern recognition, those with schizophrenia achieved scores lower than controls. Collecting other forms of data such as surveys and steps was also feasible using the same smartphone platform.

**Discussion:**

It is feasible for those with schizophrenia to use their own smartphones to complete cognitive assessments and other measures related to their mental health. While we did not investigate the correlations between these cognitive assessments and other smartphone captured metrics like step count or self-reported symptoms, the potential to longitudinally assess cognition in the context of patients' environments outside of the clinic presents unique opportunities for characterizing cognitive burden in schizophrenia.

## 1 Introduction

Schizophrenia is among the most disabling disorders in all of medicine (Haller et al., 2014). The costs to society are greater than nearly any other chronic health condition and burden to patients and family members is tremendous. Cognitive deficits involving attention and memory are core symptoms (Rajji et al., 2014) that cause functional impairment and are associated with poor clinical outcomes. Current constructs for clinically assessing cognition produce accurate and valid results but require expert clinical raters and lengthy assessment times (Reichenberg, 2010). More importantly, these current constructs offer only a single measurement in time that cannot capture the complex temporal dynamics of systems that influence cognition across patients' daily lives. Rather than ignoring this heterogeneity and complexity with a more reductionist view of cognitive deficits in schizophrenia being largely static, we propose it is possible to now quantify them through dynamic multimodal modeling.

Traditionally, cognition has been assessed in patients with schizophrenia using lengthy validated cognitive tests (Reichenberg, 2010). In 2002, the NIMH encouraged the creation of the Measurement and Treatment Research to Improve Cognition in Schizophrenia (MATRICS) initiative that developed a cognitive battery assessing cognitive domains specifically relevant to schizophrenia and related disorders (Nuechterlein et al., 2008). However, the extensive training and time needed to complete the MATRICS, as well as other similar assessments, has limited broad use in clinical settings. Recent advances in computerized assessment offer a potential solution by automating some aspects of cognitive testing in schizophrenia, for example the Brief Assessment of Cognition in Schizophrenia (BACS) (Atkins et al., 2017) can be administered and automatically scored on tablet device. Such solutions offer useful state-related assessment but do not add dynamic or network-related understanding that are more likely to correspond to the observable clinical reality, offer an explanatory framework over time, and elucidate the complex patterns of comorbidity (Öngür, 2017; Reininghaus et al., 2015). Pioneering work by Dr. Laura Germine using population level web-testing with the TestMyBrain platform (Germine et al., 2012) has also made advances in assessing cognition at scale using the NIMH Domain Criteria (RDoC) Field Test Battery Report (Passell et al., 2019).

Smartphones offer the potential for capturing such a systems view and thus more complete and continuous assessment of cognition in serious mental illnesses like schizophrenia (Depp et al., 2016). While it is well known that cognition does not exist in isolation, and that it may fluctuate with exercise, sleep, stress, environment, and social settings (Moore et al., 2017) – capturing such a dynamic and interactive model of cognition has been challenging. Using an open source and freely available smartphone tools developed by our team (Torous et al., 2016), we have previously demonstrated that it is feasible to capture real time smartphone sensor and survey data from patients with schizophrenia to model sleep (Staples et al., 2017) and functional outcomes like relapse (Torous et al., 2018a). Realizing the importance of capturing cognition, our team has since developed a suite of simple smartphone-based assessments for targeting attention and memory in schizophrenia. Our goal is not to replicate current gold standard cognitive assessments but rather to offer patients ‘games’ that likely draw upon certain cognitive domains, such as visual attention and task switching, in our modified Trails A/B Task and visuospatial working memory in our modified Spatial Span Task. Unlike traditional cognitive assessments, use of a smartphone makes it possible to record the timing related to each screen touch event and thus reproduce the process of completing the assessment (Barnett et al., 2018). At the same time it possible to capture the physical, social, psychological, and physiological state of the person completing the task through simultaneous use of passive data (either from sensors such as global positioning system (GPS) or logs such as communication logs) and concurrent active data assessments, such as a phone survey.

Clinical experience and published evidence from our team (Gay et al., 2016; Torous et al., 2018b) and many others (Lal et al., 2015; Bucci et al., 2018; Jonathan et al., 2017) confirms that patients with schizophrenia increasingly own smartphones and are willing to use applications (apps) as part of clinical care. While no app can replicate a gold standard cognitive assessment, today it is possible to capture novel micro cognitive tasks and assess their clinical relevance. While most studies assessing cognition via smartphones to date have been done with healthy controls (Dagum, 2018; Moore et al., 2017), recent studies involving patients with mental health conditions suggest promise. One study examined smartphone-based cognitive assessments in patients with substance abuse disorders over a one-week period and found that cognitive capacities varied as a function of environment, fatigue, and other factors (Bouvard et al., 2018). Another study used keyboard typing on a smartphone as a proxy for cognition in patients with bipolar disorder and found that changes in mood state were correlated with changes in phone usage (Zulueta et al., 2018). But to date no study has explored the feasibility of this approach in schizophrenia.

Understanding that performance on smartphone cognitive tasks will vary by the patients' clinical symptoms, behavioral, and social/physical environment, we hope to capture relevant data in a simple, scalable, and transparent manner. Combined with novel statistical methods, we propose it may be possible to transform this multivariate, heterogeneous, and longitudinal data into biomarkers of illness that may track with both biological mechanisms and individual-level trajectories of cognition.

## 2 Methods

Eighteen participants with schizophrenia in active treatment were recruited from an outpatient state mental health clinic in Boston and 17 healthy controls were recruited from local colleges to participate in this study (Table 1). A clinical diagnosis of schizophrenia was confirmed with the treating psychiatrists at the state mental health clinic and healthy controls were screened for lack of any current or prior mental illness using the Mini International Neuropsychiatric Interview (Sheehan et al., 1998). Inclusion criteria for those with schizophrenia included: age 18 or older, in active treatment at the state outpatient clinic, owning a smartphone able to run the study app, and ability to sign informed consent. Comorbid illness was not an exclusion factor.

**Table 1:** Demographic table.

	Healthy controls	Patients with schizophrenia	p
n	17	18	
Age (mean (sd))	23.71 (1.26)	26.06 (5.26)	0.082
Sex = male (%)	11 (64.7)	12 (66.7)	1
Race (%)			<0.001
American Indian or Alaskan Native	0 (0.0)	1 (6.2)	
Asian	14 (82.4)	0 (0.0)	
Black or African-American	1 (5.9)	4 (25.0)	
Multiracial or other	1 (5.9)	0 (0.0)	
White Caucasian	1 (5.9)	11 (68.8)	
Education (%)			0.002
4-year college graduate or higher	16 (94.1)	7 (38.9)	
High school graduate/GED	1 (5.9)	4 (22.2)	
Some college	0 (0.0)	7 (38.9)	

Inclusion criteria for study controls were: age 18 or older, no present or prior history of reported mental illness, and owning a smartphone able to run the study app. All participants signed a written informed consent and the study was reviewed and approved by both the Beth Israel Deaconess Medical Center and State of Massachusetts Department of Mental Health Institutional Review Boards.

**Fig. 1:**
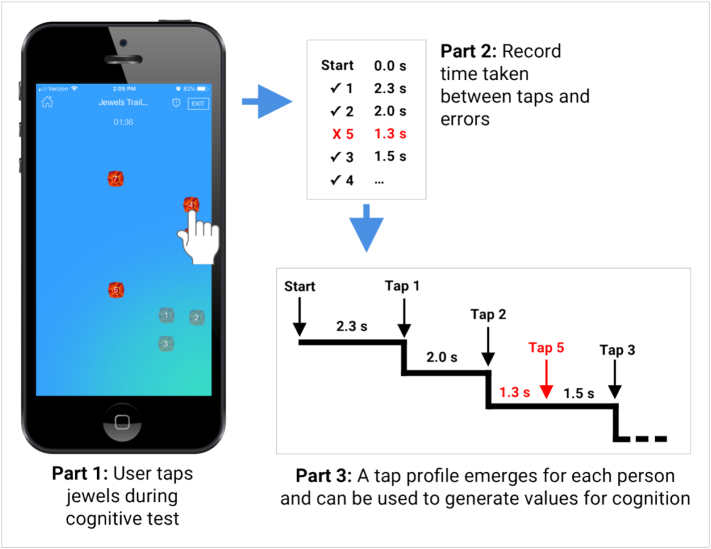
Simplified Jewels A schematic.

Participants were asked to complete two different cognitive assessments by installing and running our group's LAMP smartphone app (Torous et al., 2019) on their personal devices. Participants were prompted by an app notification to complete up to two assessments per week, as well as up to three survey assessments per week about clinical symptoms, but were free to ignore these. No payment or compensation was offered for engagement with the app. The app offered assessments inspired by the trail-making task but modified for use on a smartphone and adapted using several iterations of patient feedback to improve engagement. The resulting Jewels-A and Jewels-B assessments are similar to the classic Trails-A and Trails-B assessments in that the Jewels-A asks users to tap on numbered jewels, displayed in a random order, as quickly and accurately as possible (see Fig. 1). The Jewels-B task asks users to complete a similar assessment but with task switching as the trail must alternate between a number and a letter in the right order (1 → A → 2 → B → 3 → C etc.).

During each assessment, the app records the subject's ID, the game level, the timestamp and the item of every touch event on the screen. As shown in Fig. 1, the raw data are a temporal sequence with corresponding indicators showing whether the current touch is correct. A naive way to analyze the data is to calculate the time between two consecutive touch events and overall accuracy adjusted by the assessment level, but a meaningful assessment of cognition must simultaneously consider both aspects of performance, e.g., a longer response time usually results in better accuracy and vice versa. Furthermore, a naive quantification of either aspect in isolation neglects the sequential nature of data and does not account for the temporal ordering of events. To address these concerns, we utilized an estimation strategy that incorporated the temporal response and accuracy and is also suitable for this type of sequential data. We propose viewing the temporal sequence of assessment events as event times in survival analysis with appropriate censoring based on the game level. Using this approach, each game could be represented as a Kaplan–Meier curve and analyzed by a Cox proportional hazard model to obtain a unique estimate of the hazard ratio compared to a reference standard game.

**Fig. 2:**
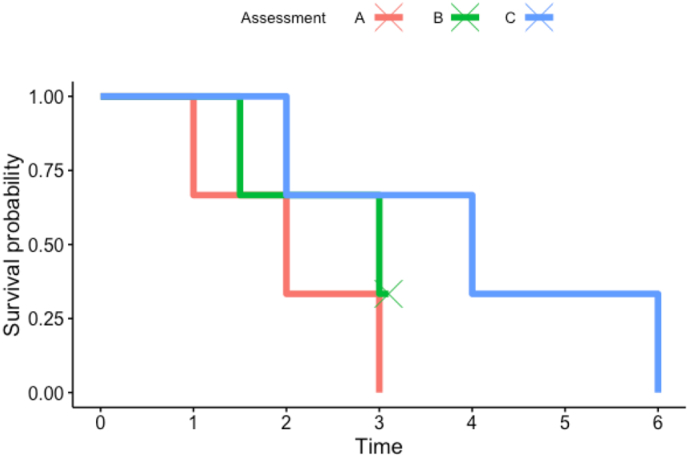
Example plot comparing survival plots.

Consider the example below in Fig. 2, representing performance on the Jewels A assessment. Assessment A is a reference path, based on average response times in controls. Assessment B and C are separate Jewels A assessments completed by the same participant. The difficulty level for each path corresponds to the number of jewels on the screen. Both the reference path and assessment C have three jewels to tap, and are difficulty level three, whereas assessment B has two jewels to tap and is difficulty level two. In this example, assessment C finishes later than the reference assessment, with longer response times for each jewel tap, a smaller hazard ratio than the reference (0.189 vs. 1) and therefore a lower cognition value. Assessment B finishes at the same time as the reference assessment but has a different difficulty level and longer response times for each tap. However, it is still comparable with the other assessments if we apply appropriate censoring and consider its hazard ratio (0.442), which is between 0.189 and 1. This ratio places the cognitive measure of assessment B between that of assessment C and that of the reference group (please see Supplementary materials for the estimates). Thus, the survival analysis enables us to compare performance on the task regardless of the number of jewels in the game.

The log hazard ratios are then adjusted by assessment levels through a linear mixed model to ensure comparisons are made against the same baseline. Finally, the adjusted log hazard ratios from each individual are summarized into a single number using inverse-variance weighting, which is the ‘beta value’ we used in this paper. The proposed method is able to differentiate patients and controls more efficiently than the naive method as demonstrated in the Results section. Please see Supplementary materials for details about the method.

## 3 Results

Over the 12-week study, the mean number of assessments completed by controls on Jewels A was 8 and Jewels B was 8. The mean number by those with schizophrenia on Jewels A was 24 and Jewels B was 22.

**Fig. 3:**
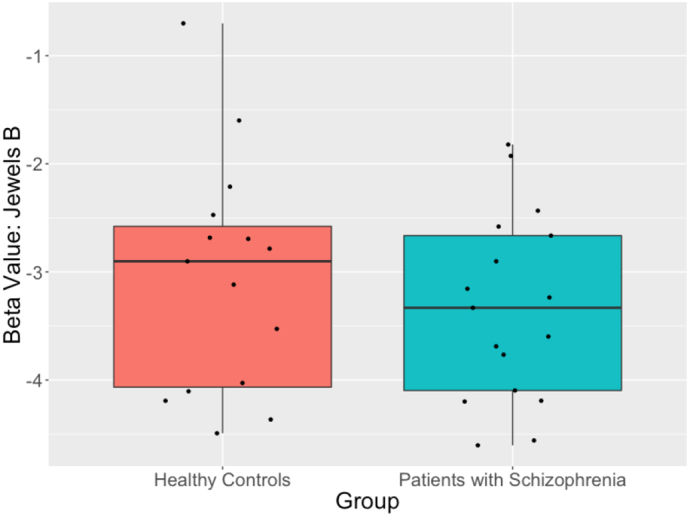
Jewels B group comparison.

Study-wide beta values for Jewels A and Jewels B were calculated for the 17 controls and 18 participants with schizophrenia. Mean beta values for Jewels A were −1.94 and −1.85 respectively and did not differ significantly (p = 0.67). Mean beta values for Jewels B were −3.08 and −3.88 respectively and did differ significantly (p = 0.0080, Fig. 3).

**Fig. 4:**
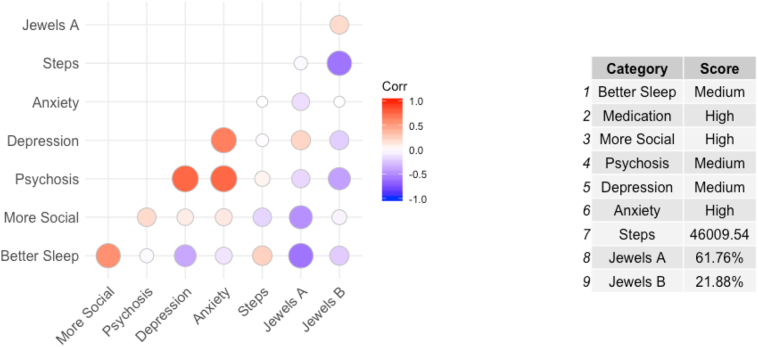
Weekly correlations in a single participant.

Weekly beta values were also computed for each participant with schizophrenia as a way to monitor individual changes in cognition relative to each participant's baseline over the course of the study. Fig. 4 shows an example of how weekly cognition could be used in subsequent analyses. In this example, we constructed a correlation matrix with multiple streams of data that can be gathered from the phone, including symptoms, cognition, and digital biomarks like step count.

## 4 Discussion

In this paper we have demonstrated the feasibility of capturing cognitive data from screen touch patterns in the context of simple smartphone assessments and this method can be used to distinguish those with schizophrenia from healthy controls. While preliminary, these results underscore the potential use of mobile monitoring to offer ecologically and longitudinally practical tools to measure and understand the interaction of mental, social, environmental, and physiological factors on cognition in serious mental illness.

Using a smartphone as a way to measure these complicated constructs presents both new challenges and opportunities. Because smartphone-based digital phenotyping enables us to capture information on when and where an assessment is taken, it enables us to capture real-time information in a person's daily life, it is now possible to ask new questions about cognition in the context of variations with sleep, physical activity, sociability, and other. As noted in Fig. 4, simple correlation plots with app-reported symptoms in addition to the cognitive assessments discussed in this paper, suggest possible clinical utility although we note such are not yet validated. More work will be needed to first validate these smartphone based cognitive assessments as well as the self-report and physical activity outcomes derived from the smartphone. But the potential to use this app to monitor relative changes in cognition and correlations with other symptoms and signs via the smartphone when a patient is starting a new antipsychotic medication are compelling.

Several next steps for utilizing this technology could be considered. First, longitudinal assessments over the course of cognitive remediation interventions while comparing to gold standard assessments (like the MATRICS) could help further establish the validity of this assessment method. Second, neuroimaging-based studies where participants complete functional cognitive tasks in the scanner (Gao et al., 2019) would further the biological understanding of the actual underlying cognitive domains assessed. Third, our result that those with schizophrenia completed more cognitive assessments than healthy controls also suggests engagement rates will differ based on the populations studied, so further collaborative design work with patients could help ensure these smartphone assessments remain engaging. While we did not compensate any participants to engage with the app in order to ensure our results are more generalizable, our results leave open the question if lack of interest from controls or more motivation from those with schizophrenia contributed to differing rates of engagement. Prior research has demonstrated that those with schizophrenia are interested in digital health tools and are willing to engage with apps they find useful (Achtyes et al., 2018; Ben-Zeev et al., 2018).

Our results are in line with prior research in using touch screens on smartphones to assess aspects of cognition. One group recently published a feasibility study of assessing cognition with smartphones in a sample of 34 outpatients over a 7-day period (Bouvard et al., 2018). Another group has also reported results of a one-week study assessing the correlation between gold standard cognitive assessments and smartphone touch screen patterns in a sample of 27 healthy adults reporting positive correlations (Zulueta et al., 2018). Our study differs from these prior efforts in that our population includes subjects with serious mental illness (e.g. schizophrenia), had a longer study duration, and our app is publicly available at: https://github.com/BIDMCDigitalPsychiatry/LAMP-app.

Several limitations warrant consideration in interpreting our results. First, further validation in an independent sample is necessary to assess the reliability of our findings. To facilitate this, we have made our source code for the LAMP smartphone app freely available in the public domain. Second, we did not compare our app-based cognitive assessments to gold standard clinical tests, as our research question and clinical use case focuses on detecting interpersonal changes in cognition relative to other symptoms and signs. That said, a better understanding of the neuroscience behind these app-based cognitive assessments is a critical next step for this work. We are currently planning such studies that will feature gold standard assessment of attention and memory. Realizing that while the trails tests do not assess all cognitive domains of relevance in schizophrenia, we have created other smartphone based cognitive assessments on the LAMP app and are currently in the processing of assessing their utility.

Third, the proposed method treats the number of mistakes as a categorical variable with “greater than one mistake” as one level, so that the method cannot tell the difference between games with a same Kaplan–Meier curve but with different number of mistakes. Fourth, the method can be computationally intensive if several weeks of data are aggregated, a weekly/monthly update is recommended for a faster runtime. Fifth, while our control sample was balanced in terms of age, it was not balanced in terms of education and this may have influenced results. As a feasibility study, college students were a practical control group to ensure results between groups did not separate on the easier cognitive task as we found, but future studies will benefit from better matching of education. Given the unmet need for college mental health service and high rates of ownership of smartphones in this population – understanding the potential of digital mental health in college students is important. Our sample did feature a larger Asian population which is notable as often this demographic is less likely to seek mental health services. Thus, these limitations in this current study offers potential for exploring new use cases in new populations.

## 5 Conclusion

Smartphone-based digital phenotyping of cognition in serious mental illnesses like schizophrenia is feasible and represents a novel approach to assess cognition in the context of everyday life. Like all new approaches, its success will depend on future research that is able to validate the pilot results presented here and clinical studies that demonstrate the utility of this information to advance care.

## Funding sources

This work was supported by a NIMH Mentored Patient-Oriented Research Career Development career training award to JT (1K23MH116130-01) and a Young Investigator grant to JT from the Brain and Behavior Research Foundation.

## Conflicts of interest

None. JT and JP have received research support from Otsuka Pharmaceuticals unrelated to this paper.

## Appendix A Supplementary data

Supplementary material Image 1
