# Supplementary material for: Assessing the potential of longitudinal smartphone based cognitive assessment in schizophrenia: A naturalistic pilot study
Source: Schizophr Res Cogn. 2019 Apr 18;17:100144. doi: 10.1016/j.scog.2019.100144 (PMC6476810; doi:10.1016/j.scog.2019.100144)
Supplement: Supplementary file 1 — Supplementary material [file mmc1.docx]

**Supplementary Materials**

1. How to transform the temporal sequence from the game to the time-to-event in the survival analysis

If we consider each game as an arm in a clinical trial and each jewel as a ‘participant’ in that arm, an event occurs whenever a user correctly touches an item. The number of ‘participants’ across all ‘arms’ is fixed at the number of jewels in the game with the highest level. If a user plays a lower level game, all the events after the last actual touch (at time T) are censored, which means the time needed from beginning until the next correct touch is greater than T if the user played the highest level game. For example, if user A plays a level 3 game with correct touches at T=[1,2,3] and user B plays a level 5 game with correct touches at T=[1,2,3,4,5], user A would spend more than 3s to correctly touch the fourth and fifth item if he played a level 5 game instead, thus the time to event in ‘arm’ A can be considered as [1, 2, 3, 3.1+, 3.2+] given the minimal time length of a touch is 0.1s.

1. Cox proportional hazard model and “pseudo” reference group

After the transformation, we take a subset of all the highest level games played by the controls and calculate the average response time per touch from those games. The average values are used to construct a ‘pseudo’ game record to function as a reference group for every participant. A Cox proportional hazard model is used to obtain a unique estimate of the hazard ratio for each game compared to the reference group.

$h(t)=h_{0}(t)\times exp(game+mistake)$,

where “game” is a factor and “mistake” is a categorical variable defined as below: mistake = 0 if the participant didn’t commit any mistake; mistake = 1 if the participant only made one mistake; and mistake = 2 if the participant committed more than one mistake.

3. Estimates in the example

We used a toy example in the paper to illustrate the concept of hazard ratio, here are more details about the data and how we estimated the hazard ratios. We assume there are three games, game 0 is a standard level 3 game with taps at time [1, 2, 3] and censoring status [1, 1, 1]; game 1 is a level 2 game with taps at time [1.5, 3, 3.1] and censoring status [1, 1, 0] (please see supplementary materials 1 to see why we transform the data in this way); game 2 is a level 3 game with taps at time [2, 4, 6] and censoring status [1, 1, 1]. As shown in supplementary materials 2, you can then fit a CoxPH model: Surv(time,status)~game_id (we don’t add mistakes in the toy example) and obtain the hazard ratios stated in the paper.

4. Linear mixed model and adjusted hazard ratios

To account for the homogeneity within the same participant, we apply a linear mixed model to find the relationship between the hazard ratio and the game level. We fit the model below:

$$\beta_{ij} = w\times level +\alpha_{i}+\epsilon_{ij},$$

where $\beta_{ij}$denotes the hazard ratio from the jth game played by the ith participant, level is a continuous variable, $\alpha_{i}$is the individual effect for participant i and $\epsilon_{ij}$is a residual.

Adjusted hazard ratios are obtained though setting the level to the highest.

A similar analysis has been done to study the relationship between the variance of hazard ratio and the game level, but the effect size can be ignored.
